# Single Administration of Melatonin Modulates the Nitroxidergic System at the Peripheral Level and Reduces Thermal Nociceptive Hypersensitivity in Neuropathic Rats

**DOI:** 10.3390/ijms18102143

**Published:** 2017-10-14

**Authors:** Elisa Borsani, Barbara Buffoli, Veronica Bonazza, Russel J. Reiter, Rita Rezzani, Luigi F. Rodella

**Affiliations:** 1 Department of Clinical and Experimental Sciences, Division of Anatomy and Physiopathology, University of Brescia, Viale Europa 11, 25123 Brescia, Italy; elisa.borsani@unibs.it (E.B.); barbara.buffoli@unibs.it (B.B.); veronica.bonazza@unibs.it (V.B.); rita.rezzani@unibs.it (R.R.); 2 Interdipartimental University Center of Research “Adaption and Regeneration of Tissues and Organs—(ARTO)”, University of Brescia, Viale Europa 11, 25123 Brescia, Italy; 3 Department of Cell Systems and Anatomy, The University of Texas Health Science Center, San Antonio, TX 78229, USA; reiter@uthscsa.edu

**Keywords:** melatonin, neuropathic pain, rats, nitroxidergic system, skin, dorsal root ganglia

## Abstract

Neuropathic pain is a severe condition with unsatisfactory treatments. Melatonin, an indolamine, seems to be a promising molecule suitable for this purpose due to its well-known anti-inflammatory, analgesic, and antioxidant effects, as well as its modulation of the nitroxidergic system. Nevertheless, the data on its mechanism of action and potentialities are currently insufficient in this pathology, especially at the peripheral level. Thus, this work evaluated the effect of a single administration of melatonin in an established mononeuropathy pain model that monitors the behaviour and the changes in the nitroxidergic system in dorsal root ganglia and skin, which are affected by nervous impairment. Experiments were carried out on Sprague Dawley rats subdivided into the sham operated (control) and the chronic constriction injured animals, a model of peripheral neuropathic pain on sciatic nerve. Single administrations of melatonin (5–10 mg/kg) or vehicle were injected intraperitoneally on the 14th day after surgery, when the mononeuropathy was established. The animals were behaviourally tested for thermal hyperalgesia. The dorsal root ganglia and the plantar skin of the hind-paws were removed and processed for the immunohistochemical detection of neuronal and inducible nitric oxide synthases. The behavioural results showed an increase of withdrawal latency during the plantar test as early as 30 min after melatonin administration. The immunohistochemical results indicated a modulation of the nitroxidergic system both at dorsal root ganglia and skin level, permitting speculate on a possible mechanism of action. We showed that melatonin may be a possible therapeutic strategy in neuropathic pain.

## 1. Introduction

The neuropathic pain, a chronic pain, is a disabling condition that may be due to an injury of central or peripheral nervous system. It affects about 1% of the population, and can be resistant to currently available analgesics. So, the disruption of a normal life is almost inevitable for such patients. To date, despite numerous efforts both within basic research and in clinical trials, the treatments are not yet satisfactory and require innovative therapeutic strategies. Such requirements could be addressed by new information on the neuronal mechanisms of this pathology. The availability of animal models for this condition has permitted us to observe related morphological and molecular alteration in both the central, [1,2,3] and the peripheral nervous systems [2,4,5,6]. Moreover, a link between the impairment of the nervous system and an alteration of skin homeostasis (morphology and expression of molecular markers involved in nociception) has been demonstrated during neuropathic pathologies [6,7]. In particular, the involvement of keratinocytes in this process was discussed, since their stimulation is sufficient to evoke acute nociception-related responses [7,8]. It is noteworthy that the keratinocyte activity is influenced by a number of signaling molecules released from nerve endings or other non-neuronal cells present in the skin [9]. In light of these data, the study on the correlation between peripheral nervous system alterations, such as dorsal root ganglia (DRG), which contain the pyrenophores of the first nociceptive neurons, and skin, which is innervated by such neurons, could be interesting and promising in this field.

New therapeutic perspectives for neuropathic pain include the evaluation of the indolamine melatonin. Melatonin is produced by the pineal gland, but also by other extra-pineal sites, such as skin [10,11,12]. It plays a fundamental role in the neuroimmunoendocrine system [13,14], but it also functions as a potent antioxidant that scavenges hydroxyl free radicals and many related reactants [15,16]. In fact, melatonin has been shown to have substantial analgesic effects [17,18,19], and its pain modulatory properties are generally recognised e.g., [20,21,22,23]. Nevertheless, the involvement of melatonin in neuropathic pain regulation is not fully understood, and is only sparingly mentioned e.g., [18,24,25,26,27]. In particular, only three of the reports regard the peripheral structures that could be promising targets for pain relief. These papers are from: Kumar and colleagues [24], who analysed the oxidative stress in the sciatic nerve; Kahya and colleagues [26], who examined apoptosis and oxidative stress in DRG; and Areti and colleagues [27], who reported the positive effect of melatonin through also examining DRG as well as skin. Melatonin exhibits its antioxidant properties through binding to their specific receptors or through a receptor independent action. In the first case, it directly can bind four different receptor subtypes. Two of them are membrane-associated receptors, while the other two are intracellular receptors, the quinine oxidoreductase-2, or MT3, and the nuclear receptor, RORα (retinoid-related orphan receptor α). Membrane melatonin receptors are classified based upon their kinetic properties and pharmacological profiles into MT1 (Mel 1a) and MT2 (Mel 1b) melatonin receptor subtypes [28], which have been identified in nervous system structures involved in nociceptive transmission [29,30,31]. In keratinocytes, the expression of MT1, MT2, MT3, and RORα (reviewed in [32]) has been observed. In particular, melatonin-induced antinociception appears to be mediated via specific interaction with melatonin receptors [33], although there are probably also effects on other signalling systems, such as modulation of the N-methyl D-aspartic acid (NMDA) receptor function [34]. Moreover, in different animal models of neuropathic pain [24,26,35], the administration of melatonin reduces oxidative damage and it is partially reversed by L-arginine, which suggests that nitric oxide synthase (NOS) is involved in this response. Nitric oxide (NO) is a free radical, but also an important signaling molecule produced in the neurons mainly by two isoforms of NOS: a constitutive (neuronal) isoform (nNOS), and an inducible isoform (iNOS). The latter is activated in pathological conditions, and causes the prolonged synthesis of a highly uncontrolled quantity of NO that could be toxic for cells. These two isoforms participate in different ways in nociception [2]. Also, the keratinocytes express NOS and appear capable of releasing NO [36].

On the basis of these findings, we evaluated the effects of a single administration of melatonin using two doses of this indolamine (5 and 10 mg/kg,) in a neuropathic pain model on sciatic nerve (chronic constriction injury—CCI) at 14 days after surgery. Moreover, we monitored the thermal hyperalgesia at different time points and the changes in the nitroxidergic system in DRG and hind-paw plantar skin. The results showed the crucial role of melatonin on the nitroxidergic system at these anatomical sites, and permitted us to speculate on a possible mechanism of action. 

Thus, we suggest that melatonin is an alternative strategy for improving neuropathic pain.

## 2. Results

### 2.1. Behavioural Test: Thermal Hyperalgesia

**Figure 1:**
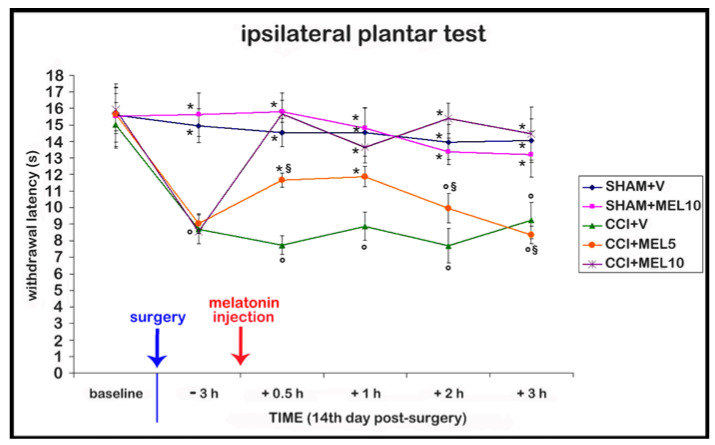
Behavioural test. Time course of the effect of different doses of melatonin (5 and 10 mg/kg) on thermal hyperalgesia evaluated as withdrawal latency to heat; this was measured by the plantar test on the right hind-paw and expressed in seconds (s). The administration of melatonin was performed as a single intraperitoneal injection at the 14th day after surgery, when the neuropathy was established. Sham-operated rats treated with 1% ethanol in saline (vehicle for melatonin) (SHAM + V); sham-operated rats treated with melatonin (10 mg/kg) (SHAM + MEL10); Chronic constriction injury (CCI) rats treated with 1% ethanol in saline (vehicle for melatonin) (CCI + V); CCI rats treated with melatonin (5 mg/kg) (CCI + MEL5); CCI rats treated with melatonin (10 mg/kg) (CCI + MEL10). Data represent means ± SEM, ° *p* < 0.05 vs. SHAM + V, * *p* < 0.05 vs. CCI + V, ^§^ *p* < 0.05 vs. CCI + MEL10.

Before the onset of the surgical procedures, no differences were detected among groups. After the surgical procedure, no differences in contralateral side were detected compared with sham-operated rats treated with vehicle 1% ethanol in saline (vehicle for melatonin), so we reported only the results on the ipsilateral side. Moreover, we confirmed that the CCI model induced thermal hyperalgesia at the 14th day, since the withdrawal latency response to the thermal stimulus of CCI rats before treatments (intraperitoneal injection of melatonin or its vehicle) is reduced compared with sham-operated rats. In addition, the posture of the CCI rats was compromised, with the foot being held in an “antalgic posture” in which the glabrous sole of the ipsilateral hind-paw was not completely placed on the floor. Furthermore, we observed that the treatment with melatonin at both doses tested (5 and 10 mg/kg), and at 0.5 and 1 h after melatonin administration, led to an improvement of thermal hyperalgesia compared with CCI rats injected with 1% ethanol in saline (vehicle for melatonin). In particular, the best effect was observed with the dosage of 10 mg/kg, which was able to reduce hyperalgesia also at 2 and 3 h after melatonin administration; these rats had also the glabrous sole of the ipsilateral hind-paw placed more firmly on the surface compared with the rats treated with the dosage of 5 mg/kg. The sham-operated animals treated with melatonin 10 mg/kg showed no significant differences compared with sham-operated animals treated with 1% ethanol in saline (vehicle for melatonin), indicating the absence of side effects such as sedation, in accordance also with the observations of Ulugol and colleagues [35]. The data are summarized in Figure 1.

### 2.2. nNOS and iNOS Immunohistochemistry

The changes on immunopositivity were only detected on the ipsilateral side. In fact, in contralateral DRG and skin, no differences were apparent with respect to the sham-operated rats treated with 1% ethanol in saline (vehicle for melatonin).

#### 2.2.1. Dorsal Root Ganglia (DRG) Small Neurons

In DRG samples, only small size neurons have been analysed, because of their primarily involvement in nociceptive transmission [37,38].

##### nNOS Staining

**Figure 2:**
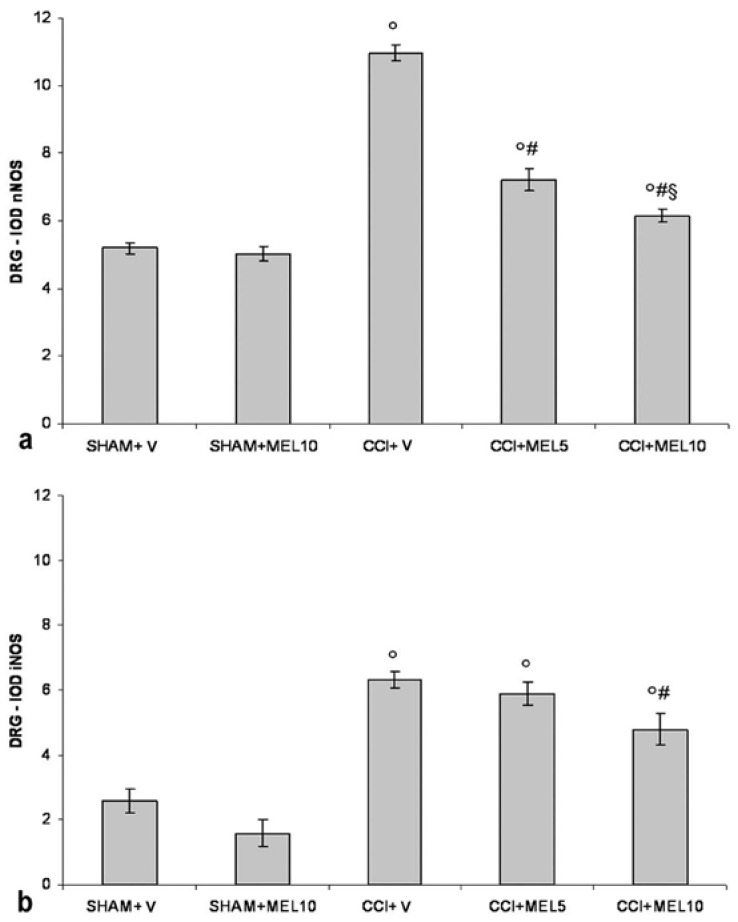
Constitutive (neuronal) isoform (nNOS) and inducible isoform (iNOS) evaluation in dorsal root ganglia (DRG). (**a**) Quantitative evaluation of nNOS immunopositivity in DRG (small size neurons − diameter < 30 µm) as IOD (integrated optical density) in the experimental animals: sham-operated rats treated with 1% ethanol in saline (vehicle for melatonin) (SHAM + V); sham-operated rats treated with melatonin (10 mg/kg) (SHAM + MEL10); CCI rats treated with 1% ethanol in saline (vehicle for melatonin) (CCI + V); CCI rats treated with melatonin (5 mg/kg) (CCI + MEL5); CCI animals treated with melatonin (10 mg/kg) (CCI + MEL10). Data represent means ± SEM, ° *p* < 0.05 vs. SHAM + V, ^#^ *p* < 0.05 vs. CCI + V, ^§^ *p* < 0.05 vs. CCI + MEL5. (**b**) Quantitative evaluation of iNOS immunopositivity in dorsal root ganglia (DRG) (small size neurons − diameter < 30 µm) as IOD (integrated optical density) in the experimental animals: sham-operated rats treated with 1% ethanol in saline (vehicle for melatonin) (SHAM + V); sham-operated rats treated with melatonin (10 mg/kg) (SHAM + MEL10); CCI rats treated with 1% ethanol in saline (vehicle for melatonin) (CCI + V); CCI rats treated with melatonin (5 mg/kg) (CCI + MEL5); CCI animals treated with melatonin (10 mg/kg) (CCI + MEL10). Data represent means ± SEM, ° *p* < 0.05 vs. SHAM + V, ^#^ *p* < 0.05 vs. CCI + V.

**Figure 3:**
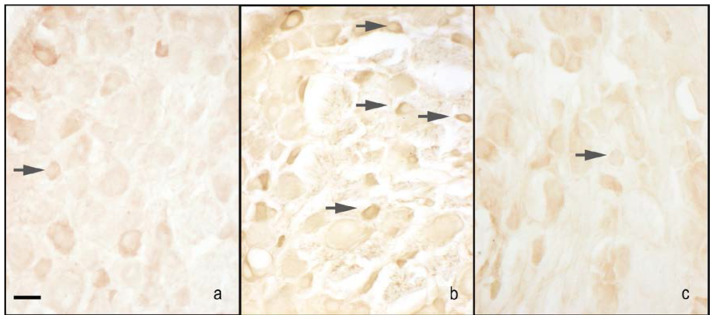
Microphotographs of nNOS immunostaining of the DRG. Microphotographs of nNOS immunostaining of the right DRGs of sham-operated rats treated with 1% ethanol in saline (vehicle for melatonin) (**a**), CCI animals treated with 1% ethanol in saline (vehicle for melatonin) (**b**) and CCI animals treated with melatonin 10 mg/kg (**c**). Arrows indicate small neurons. Bar = 40 μm.

The nNOS immunoreactivity was localised in the cytoplasm of small size neurons, and the nuclei were virtually unstained. The CCI rats treated with 1% ethanol in saline (vehicle for melatonin) had an increase of staining compared with the sham-operated rats treated with 1% ethanol in saline (vehicle for melatonin). Treatment with melatonin (5 and 10 mg/kg) induced a decrease of staining in CCI rats, and the effect was particularly relevant with the highest dosage used. Moreover, the treatment with melatonin at 10 mg/kg did not affect nNOS immunopositivity in sham-operated rats compared with the administration on 1% ethanol in saline (vehicle for melatonin) (Figure 2a and Figure 3).

##### iNOS Staining

**Figure 4:**
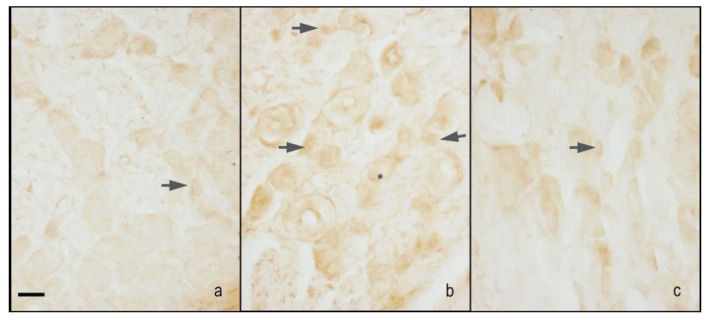
Microphotographs of iNOS immunostaining of the DRG. Microphotographs of iNOS immunostaining of the right DRGs of sham-operated rats treated with 1% ethanol in saline (vehicle for melatonin) (**a**), CCI animals treated with 1% ethanol in saline (vehicle for melatonin) (**b**), and CCI animals treated with melatonin 10 mg/kg (**c**). Arrows indicate small neurons. Bar = 40 μm.

The iNOS immunoreactivity was localised in the cytoplasm of small size neurons, and the nuclei were virtually unstained. The CCI rats treated with 1% ethanol in saline (vehicle for melatonin) had an increase of staining compared with sham-operated rats treated with 1% ethanol in saline (vehicle for melatonin) and sham-operated rats treated with melatonin 10 mg/kg. Treatment with melatonin 10 mg/kg, but not 5 mg/kg, induced a decrease of staining in CCI rats. Moreover, the treatment with melatonin at 10 mg/kg did not affect iNOS immunopositivity in sham-operated rats compared with the administration on 1% ethanol in saline (vehicle for melatonin) (Figure 2b and Figure 4).

#### 2.2.2. Hind-Paw Skin Epidermis

An important observation on skin morphology was the reduction of epidermal thickness in CCI rats treated with 1% ethanol in saline (vehicle for melatonin) compared with the sham-operated rats. This indicates a “suffering” tissue as a consequence of nerve damage, as reported by Kojundzic and colleagues [39].

##### nNOS Staining

**Figure 5:**
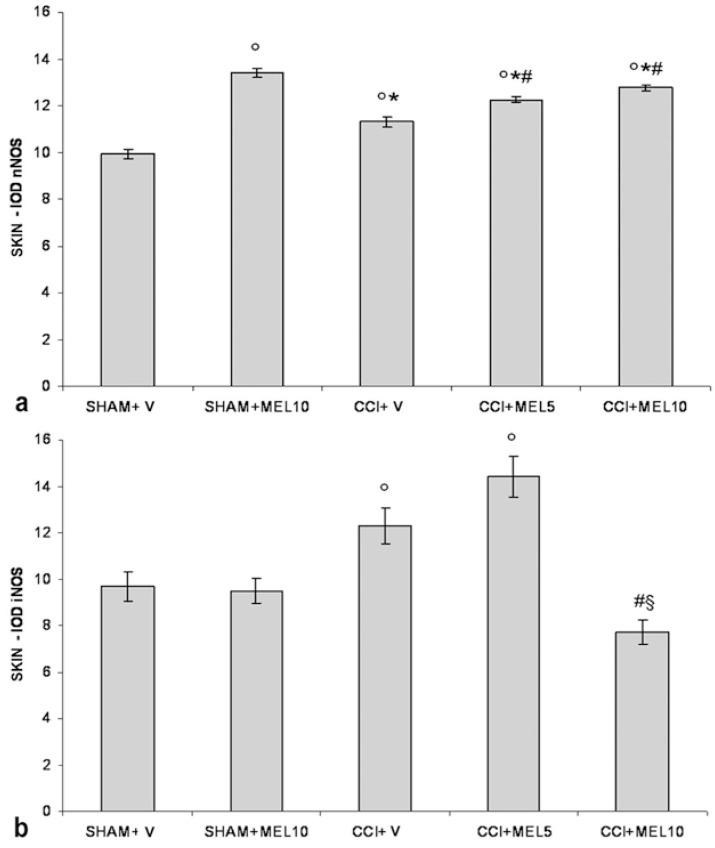
nNOS and iNOS evaluation in plantar skin. (**a**) Quantitative evaluation of nNOS immunopositivity in the epidermis of the right hind-paw plantar skin as IOD (integrated optical density) in the experimental animals: sham-operated rats treated with 1% ethanol in saline (vehicle for melatonin) (SHAM + V); sham-operated rats treated with melatonin 10 mg/kg (SHAM + MEL10); CCI rats treated with 1% ethanol in saline (vehicle for melatonin) (CCI + V); CCI rats treated with melatonin (5 mg/kg) (CCI + MEL5); CCI rats treated with melatonin (10 mg/kg) (CCI + MEL10). Data represent means ± SEM, ° *p* < 0.05 vs. SHAM + V, * *p* < 0.05 vs. SHAM + MEL10, ^#^ *p* < 0.05 vs. CCI + V. (**b**) Quantitative evaluation of iNOS immunopositivity in the epidermis of the right hind-paw plantar skin as IOD (integrated optical density) in the experimental animals: sham-operated rats treated with 1% ethanol in saline (vehicle for melatonin) (SHAM + V); sham-operated rats treated with melatonin 10 mg/kg (SHAM + MEL10); CCI rats treated with 1% ethanol in saline (vehicle for melatonin) (CCI + V); CCI rats treated with melatonin (5 mg/kg) (CCI + MEL5); CCI rats treated with melatonin (10 mg/kg) (CCI + MEL10). Data represent means ± SEM, ° *p* < 0.05 vs. SHAM + V, ^#^ *p* < 0.05 vs. CCI + V, ^§^ *p* < 0.05 vs. CCI + MEL5.

**Figure 6:**
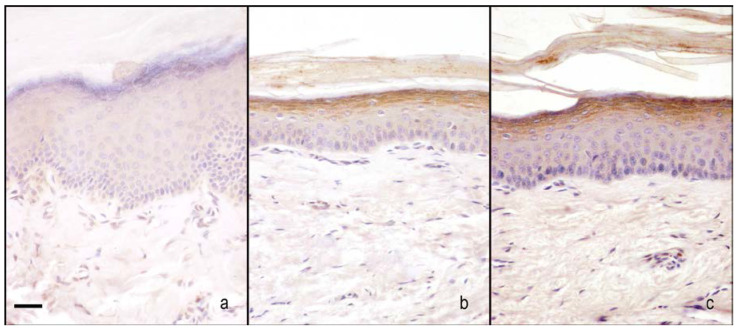
Microphotographs of nNOS immunostaining in plantar skin. Microphotographs of nNOS immunostaining in the epidermis of the right hind-paw plantar skin of sham-operated rats treated with 1% ethanol in saline (vehicle for melatonin) (**a**), CCI rats treated with 1% ethanol in saline (vehicle for melatonin) (**b**), and CCI rats treated with melatonin 10 mg/kg (**c**). Bar = 20 μm.

nNOS immunoreactivity was localised in the cytoplasm of the keratinocytes. In sham-operated rats treated with 1% ethanol in saline (vehicle for melatonin), the nNOS appeared moderately and homogeneously stained across the full thickness of the epidermis. The CCI rats treated with 1% ethanol in saline (vehicle for melatonin) had an increase of staining compared with sham-operated rats treated with 1% ethanol in saline (vehicle for melatonin); in particular, the staining ranged from moderate to high intensity, appearing more visible in the granular/translucent layers with respect to the basal and spinous layers. The administration of melatonin increased the staining in CCI rats (5 and 10 mg/kg) that maintained the same distribution pattern and even more in sham-operated rats treated with 10 mg/kg (Figure 5a and Figure 6).

##### iNOS Staining

**Figure 7:**
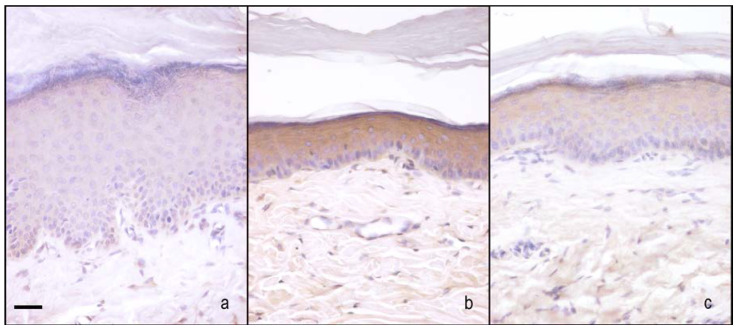
Microphotographs of iNOS immunostaining in plantar skin. Microphotographs of iNOS immunostaining in the epidermis of the right hind-paw plantar skin of sham-operated rats treated with 1% ethanol in saline (vehicle for melatonin) (**a**), CCI rats treated with 1% ethanol in saline (vehicle for melatonin) (**b**), and CCI rats treated with melatonin 10 mg/kg (**c**). Bar = 20 μm.

iNOS immunoreactivity was localised in the cytoplasm of the keratinocytes. In sham-operated rats treated with 1% ethanol in saline (vehicle for melatonin), iNOS appeared moderately and homogeneously stained across the full thickness of the epidermis. The administration of melatonin (10 mg/kg) did not alter the basal staining intensity. The CCI rats treated with 1% ethanol in saline (vehicle for melatonin) had a great increase in staining compared with these two last groups. It appeared dark brown and homogenous among keratinocytes across the full thickness of the epidermis. The administration of 5 mg/kg melatonin did not change this situation, while the administration of higher dose melatonin (10 mg/kg) greatly reduced the staining intensity (Figure 5b and Figure 7).

## 3. Materials and Methods

### 3.1. Animal Treatment

Experiments were carried out on 25 Sprague Dawley male rats (200 g BW, Harlan, Italy). The animals were housed in cages (two or three animals/cage) with food and water ad libitum and kept in an animal house at a constant temperature of 20 °C with 12 h alternating light–dark cycle, to minimise the circadian variations. Before the beginning of the experiments, the rats were left housed in the animal facility for at least one week.

All efforts were made to minimise animal suffering and the number of animals used.

All of the experimental procedures were approved by Animal Care and Use Committee of the Italian Ministry of Health (105/2011-B-31/05/2011) and comply the commonly accepted “3Rs” indication.

The chronic constriction injury (CCI) model of mononeuropathy is assessed through placing around the sciatic nerve four ligatures, which causes a constriction without damaging nerve fibres, and so permitting the transport of their inputs into the laminae I–IV of the dorsal horn of the spinal cord [40,41].

In the present study, the rats were anesthetised by intraperitoneal injection of Zoletil (60 mg/kg i.p.—Virbac, France), and the right sciatic nerve was exposed at the level of the mid-thigh by blunt dissection and separated from the adhering tissue immediately proximal to its trifurcation. Four ligatures were loosely tied around the nerve (2 Ph. Eur., MERSILK, Ethicon; Johnson and Johnson, Belgium) at 1–2 mm distance according to the method described by Bennett and Xie [42]. However, the sham-operated rats had the right sciatic nerve exposed at the same level, without ligature, and so serve as controls.

The treatment with melatonin, in combination with the CCI procedure, was administered in two different dosages of 5 mg/kg and 10 mg/kg, according to Laurido and colleagues [34] and Kahya and colleagues [26]. Melatonin (Sigma-Aldrich, Milan, Italy) was dissolved in ethanol and subsequently in saline to obtain a final concentration of ethanol of about 1% [43]. The administration was performed as a single intraperitoneal injection (100 µL/150 g) at the 14th day after surgery, when the neuropathy was established. In addition to the groups identified above, the current study also assessed sham-operated and CCI rats intraperitoneally injected with 1% of ethanol dissolved in saline (vehicle of treatment with melatonin), and sham-operated animals treated with melatonin at a final dose of 10 mg/kg.

The animals of all of the experimental groups were monitored for thermal hyperalgesia. To standardise the time of the sacrifice after the behavioural test, which took place 4 h after injection, all rats were anaesthetised with Zoletil (60 mg/kg i.p., Verbatic, France) and transcardially perfused with saline followed by 1 L of 4% paraformaldehyde in phosphate buffer saline (0.1 M, pH 7.4).

After fixation, the plantar skin of the hind-paws and dorsal root ganglia (DRG) L4–L5–L6, corresponding to the sciatic afferent fibres were carefully removed.

### 3.2. Behavioural Test: Thermal Hyperalgesia

The current study uses a thermal stimulus to evaluate hyperalgesia. Responses to thermal stimuli of all of the animals were measured at baseline (before the surgical procedure), 3 h before, and 0.5, 1, 2, 3 h after melatonin or vehicle administration. Measurements were performed on both the ipsilateral and contralateral hind-paws of all the rats by researchers who were blind to the animal group.

Thermal hyperalgesia was tested according to the Hargreaves procedure [44] using a plantar test apparatus (Ugo Basile, Comerio, Italy). Briefly, rats were placed in clear plexiglass cubicles and allowed to acclimatise. A constant intensity radiant heat source (beam diameter 0.5 cm and intensity 40 I.R.) was aimed at the mid-plantar area of the hind-paw.

The time, in seconds (s), from initial heat source activation until paw withdrawal was recorded four times. The data of all of the animals were analysed and compared by two-way ANOVA with repeated measures in one factor followed by Tukey’s test. The level of significance was set at 5% (*p* < 0.05).

### 3.3. Immunohistochemical Evaluations

The plantar skin of the hind-paws and dorsal root ganglia (DRG) L4–L5–L6 were post-fixed in 4% paraformaldehyde in phosphate-buffered saline (PBS) respectively for 4 h and 2 h.

The skin was embedded in paraffin wax according to standard procedures, while the DRG were cryoprotected overnight in 30% sucrose at 4 °C, and afterwards stored at −20 °C. The skin was serially sectioned at 7 µm by microtome (Microm HM 325), while the DRG were serially sectioned at 35 µm using a cryostat (Leica 1900) and collected in tris-buffered saline (TBS). Only the wax sections were deparaffinised, rehydrated, and subjected to antigen retrieval in 0.05 M sodium citrate buffer (pH 6.0) in a hot water bath (98 °C for 20’) before the immunohistochemical procedure. 

Briefly, the sections were incubated firstly in normal goat serum (10% in TBS plus 0.1% Triton X-100) for 60 min, and then in rabbit polyclonal primary antiserum directed against nNOS and iNOS (Santa Cruz Biotechnology, Santa Cruz, CA, USA) diluted, respectively, at 1:200 and 1:500 in TBS containing 3% normal goat serum and 0.1% Triton X-100, for 24 h at 4 °C. After incubation in the primary antiserum, the sections were sequentially incubated with appropriated biotinylated secondary antibodies and an avidin–biotin peroxidase complex (Vector Labs., Burlingame, CA, USA). The reaction product was visualised using hydrogen peroxide and diaminobenzidine (Sigma, St. Louis, MO, USA) as chromogen.

The immunohistochemical control was performed by omitting the primary antibody and incubating the sections with non-immune rabbit serum and with isotype-matched irrelevant rat IgGs as negative control.

The nNOS and iNOS immunopositivity was evaluated quantitatively in the epidermis of skin, and in DRG samples. In the nervous samples, only small sized neurons, characterised by a diameter of less than 30 µm [45], were analysed, since the sensory nerve terminals in the skin are the peripheral processes of small-diameter DRG neurons and primarily involved in nociceptive transmission [37,38]. Moreover, only neurons with a clearly visible nucleus were considered, and the morphological identification and evaluation was performed according to previous studies [4,36,46].

For the analysis, the immunopositivity was evaluated at a final 200× magnification using an optical microscope (Olympus, Hamburg, Germany). Digitally fixed images were analysed using an image analyser (Image Pro-Plus, Milan, Italy) by researchers unaware of the group assignment and were calculated as integrated optical density (IOD) according to Borsani and colleagues [47]. The analysis has been performed on five sections for each sample, evaluating six random fields with the same area for each section. The data collected were analysed by one-way ANOVA followed by the Bonferroni test. The level of significance was set at 5% (*p* < 0.05).

## 4. Discussion

The CCI model of mononeuropathy is a useful tool to study neuropathic pain. We evaluated the effect of a single administration of melatonin at two different dosages (5 and 10 mg/kg) that improve pain behaviour. We tested the thermal hyperalgesia, with the principal aim to use it as behavioural indicator for the subsequent immunohistochemical analysis for iNOS and nNOS, but also to minimise the stress and suffering of the animals according with the observations of Ulugol and colleagues [35]. In fact, they analysed both the thermal hyperalgesia and the mechanical allodynia in a mice neuropathic pain model after intraperitoneal treatment with high doses of melatonin (30, 60, 120 mg/kg, i.p.). Their results showed that thermal hyperalgesia decreased, while none of the doses was able to influence the response of the animals at the test for mechanical allodynia. Nevertheless, in another study by using a rat model of neuropathic pain (i.e., ligation of L5/L6 spinal nerves) and different ways of administration (intrathecal, 3–100 µg or oral, 37.5–300 mg/kg), the melatonin was able to decrease tactile allodynia [48]. Mechanical allodynia and thermal hyperalgesia are suggested to be mediated through separate mechanisms and neuronal pathways, and the drugs may affect these manifestations conversely. So, the alleviation of mechanical allodynia/hyperalgesia using melatonin cannot be completely excluded, and should be better investigated. Furthermore, these experiments suggest that the way of administration seems to be an important factor to obtain a decrease of both heat hyperalgesia and mechanical allodynia/hyperalgesia. To date, other experiments are needed to clarify the potentiality of melatonin in the neuropathic symptoms and consider the possible implications and limitations in the clinical practice.

The choice of the useful dose of melatonin for neuropathic pain is an open question. In our experiments, we chose the doses on the basis of the observations of Laurido and colleagues [34], who reported that intraperitoneally-administered melatonin induced a dose-dependent reduction of spinal wind-up using the doses of 1.25, 2.5, 5, 10 mg/kg, and that the highest dose of the drug completely depressed the C reflex gain. Our data are in agreement with these results. In fact, we observed a dose-dependent increase in withdrawal latency, and the dose of 10 mg/kg was more effective than 5 mg/kg melatonin reaching values similar to sham-operated rats treated with 1% ethanol in saline (vehicle for melatonin). Moreover, the effect of the lower dose (5 mg/kg) decreased over the time, and was no longer apparent at 3 h; on the other hand, the effects of 10 mg/kg persisted at 3 h. These results only partially agree with observation of Yu and colleagues [33]. In fact, they reported that in rats, after intraperitoneally melatonin injections at various doses (30, 60, 120 mg/kg), the anti-nociceptive effect peaked at 30 min, and continued for 100 min. To better understand these data, it is important to remember that the dose for a single intraperitoneal administration is about 100 mg/kg [35,49], while lower doses are used for daily administration for about 2–4 weeks (2.5, 3, 5, 10 mg/kg) [24,26,27]. Furthermore, clinical trials on neuropathic patients evaluating the effect of melatonin, to our knowledge, have not been published. Only few studies were carried out to analyse the effects of melatonin on post-operative pain or during chemotherapy-induced neuropathy (e.g., [50,51,52]). In these cases, the complete resolution of pain is hardly seen in humans, where the experimental conditions are influenced by different variables, and the clinical personal history of the patient is one of them. In addition, to this day, the exact time, dose and way of administration in clinical practice are still undetermined and under debate [53]. Another point of interest could be the intraplantar administration of melatonin in neuropathic pain models, even if it is not usually performed. Nevertheless, in some experimental works of local acute inflammation models (carrageenan, zymosan, formalin, lipopolysacharides, glutamate) [23,54,55,56,57], the local administration of melatonin has been investigated. These findings support the view that melatonin exerts an anti-inflammatory effect, which may be partially related to the modulation of the nitroxidergic pathway. So, the local administration should be also considered in pain therapy, even if we think that the systemic administration may ensure also a protective and antioxidant effect at all levels of the nervous system, thus supporting the compromised structure causing neuropathies.

The study of nNOS and iNOS expression permitted us to evaluate the influence of melatonin on the nitroxidergic system in peripheral structures during nociceptive transmission. Regarding the role of NO, it is related to the site of action. In fact, it could have either a pro- or anti-nociceptive action [58]. The expression of nNOS and iNOS at the DRG level in this neuropathic pain model has been previously published by our research group [36,59]; that study showed a time-increase of these molecules during the 14 days post-surgery, and so addressed their involvement at peripheral level. Moreover, other our previous experiments revealed that, after an acute nociceptive event, the time course of nNOS and iNOS over-expression detected in immunohistochemistry is over 24 h, with a clear detectable alteration that remains between the third and the sixth hour [60]. At the DRG level, we observed that melatonin, especially at the highest dose, decreased nNOS and iNOS expression in small size neurons that were considered to be nociceptive. The ability of melatonin to influence nitroxidergic system is supported by a work in which the beneficial effect of melatonin in neuropathic mice was partially reversed by L-arginine (a NO precursor) administration, suggesting that NOS are involved in this response [35]. Regarding epidermis, we observed an increase of nNOS expression in sham-operated animals treated with melatonin (10 mg/kg), while iNOS was unaffected. The increase of nNOS was observed also in chronic constriction-injured animals treated with vehicle alone, just as iNOS. In the skin, NO may have a protective and anti-nociceptive role [36,61,62]. So, we can hypothesise that melatonin modulates NO production to promote skin proliferation and decrease ROS, which is consistent with the beneficial effects in aging skin [63]. In particular, melatonin induces a rise in the mRNA expression of nNOS at nanomolar or subnanomolar concentrations [64] in HaCat cells in culture (a human keratinocyte cell line). The upregulation by melatonin of the nNOS mRNA expression appeared specific, since iNOS expression was insensitive to melatonin, as reported by others [65]. This suggests the presence of a specific receptor-mediated pathway in the rat, as already has been reported in the mouse [66].

**Figure 8:**
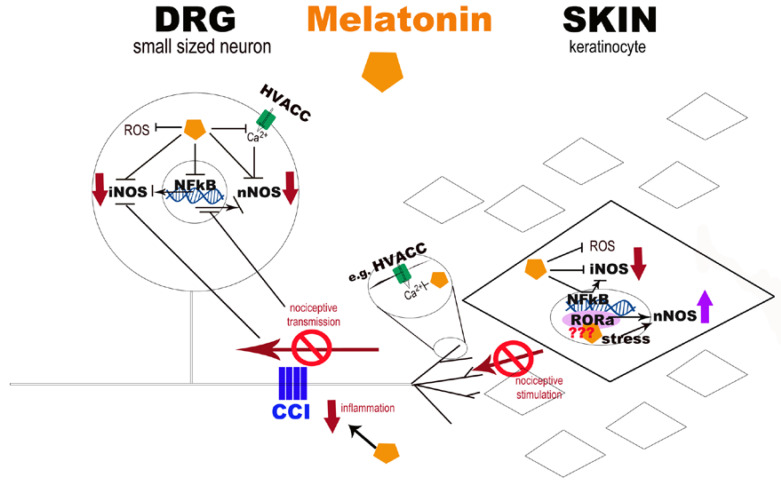
Schematic diagram of potential melatonin pathways at the skin and DRG levels. The diagram represents the potential pathways undergoing the beneficial effect of melatonin in neuropathic pain conditions at skin and DRG levels. Melatonin is highly lipid soluble, and readily crosses the plasma membrane to enter the cell; it has access to cytosolic, mitochondrial, and nuclear compartments. Through its radical scavenging action, it protects the cells from free radicals, such as nitric oxide (NO) and reactive oxygen species (ROS), and decreases the neurotransmitters/neuromodulators production, which leads to the nerve fibres excitation. So, the beneficial effect of melatonin at the skin level could also affect the sensory nerve terminals influencing the nociceptive transmission. Melatonin may also inhibit directly the catalytic activity of nitric oxide synthase (NOS) and modulate NOS expression. In particular, iNOS expression is reduced through the inhibition of the transcription factor nuclear factor-kappa B (NF-κB), both at the DRG and in keratinocytes. Regarding nNOS, at the skin level, we presume that its increased expression in sham-operated animals treated with melatonin (10 mg/kg) may be probably due to the melatonin interaction with RORα receptor, even if it has not yet been identified in the rat, and only in the mouse. At the DRG level, nNOS upregulation seems to represent a general response of neuronal cells to stress, including nerve injuries, as in chronic constriction injury model (CCI). Moreover, melatonin may reduce the intracellular calcium rise by inhibiting, for example, high voltage activated calcium channels (HVACC), and thereby inhibiting also nNOS activation.

The molecular mechanisms underlying the functional effects of melatonin remains only partly understood. In Figure 8, we propose a possible role of melatonin in NO modulation. First, both at the skin and at DRG level, melatonin protected the cell membrane, organelles, and core against free-radical damage [66] through its radical scavenging action [67,68] and its strong anti-apoptotic signalling function [15]. Melatonin is highly lipid soluble and readily crosses the plasma membrane to enter the cell; it has access to cytosolic, mitochondrial, and nuclear compartments. So, the beneficial effect of melatonin at the skin level could positively influence the nociceptive transmission, with a decrease of neurotransmitters/neuromodulators leading the nerve fibre excitation. In fact, sensory nerve terminals in the skin are the peripheral processes of small-diameter DRG neurons [37,38], and neuropathy is caused also by selective damage to C and Aδ fibres (which are the main peripheral transmitters of nociception).

Melatonin-induced anti-nociception firstly appears to be mediated via specific interaction with melatonin receptors [33]. There are probably also effects on other signalling systems, such as the modulation of the N-methyl D-aspartic acid (NMDA) receptor function [34,69] or by inhibiting high voltage activated calcium channels (HVACC) [70]. A report describing the modulation of lipopolysaccharide-induced hyperalgesia by melatonin suggested that another mechanism of action could be via the inhibition of the sensitising actions of pro-inflammatory cytokines on nociceptive sensory neurones [23]. Melatonin may also inhibit directly the catalytic activity of NOS [71,72] and the modulation of NOS expression. In fact, it reduced the expression of iNOS through the inhibition of the transcription factor nuclear factor-kappa B (NF-κB) [73], since this transcription factor is involved in iNOS expression [74,75]. This effect was also confirmed by experiments on acute exercise in rat muscle, where it prevented oxidative stress, NF-κB activation, and iNOS over-expression [76]. Regarding nNOS, its upregulation may, in most cases, represent only one component of the normal cellular stress response, resulting in cellular damage and apoptosis. In neuronal cells, for example, this is due to a large array of physical, chemical, and biological agents such as heat [77] or formalin exposure [78]. A similar response is observed after mechanical or pathological lesions, including in the spinal cord and in axonal or nerve injuries [36,56,79]. Finally, nNOS expression also appears to be regulated by changes in neuronal activity [80].

At the skin level, we presumed that the increased expression of nNOS in sham-operated animals treated with melatonin (10 mg/kg) was probably due to interaction with the RORα receptor, a possible nuclear melatonin binding site, even if it has not been identified in the rat, only in the mouse [81]. At the DRG level, melatonin may inhibit intracellular calcium rise by inhibiting HVACC in cultured rat DRG small neurones [70], and thereby inhibiting also nNOS activation; these neurons are emerging as especially attractive targets for the treatment of pain [82]. However, there is no evidence that melatonin interacts with these receptors or directly effects calcium channels.

The results presented here should be viewed as hypothesis-generating, and further study as the next step in this line would be regarding perspectives such as monitoring the role of NF-κB, HVACC, and the RORα receptor, as well as evaluating the local administration of melatonin, which all should be promising in future research. Moreover, the number of animals and the use of only male animals are of course strong limitations in this research. Many laboratory studies on human subjects have described sex differences in sensitivity to noxious stimuli, suggesting that biological mechanisms underlie such sensitivities [83]. In addition, sex hormones influence pain sensitivity. In particular, pain threshold and pain tolerance in women vary with the stage of the menstrual cycle. Variability in pain sensitivity, especially during the estrous cycle, has been demonstrated also in rodents, with the highest pain threshold occurring when progesterone levels were highest [84]. So, in pain animal experiments, the male animals are usually preferred, but this choice represents a limitation in understanding of pain mechanisms.

## 5. Conclusions

Single melatonin administration in an established mononeuropathy pain model reduces thermal hyperalgesia through the modulation of nitroxidergic system. These are the first data suggesting the use of low doses, lower than about 100 mg/kg [35,49]. Moreover, the nitroxidergic system is primarily involved in nociception, and is poorly investigated at the peripheral level. Our data underline the importance of its modulation to inhibit nociceptive transmission at the DRG, but also at the skin level, giving rise to a possible mechanism of action of melatonin at these sites.

Clinical trials on neuropathic patients evaluating the effect of melatonin, to our knowledge, have not been published, even if, in the literature, some applications on post-operative pain or during chemotherapy-induced neuropathy have been reported (e.g., [50,51,52]). Moreover, in this field, the clinical use of melatonin and their dosage is under debate (reviewed in [53]). Collectively, our results encourage the onset of clinical trials.
