# Correction: Borsani et al. Single Administration of Melatonin Modulates the Nitroxidergic System at the Peripheral Level and Reduces Thermal Nociceptive Hypersensitivity in Neuropathic Rats. *Int. J. Mol. Sci.* 2017, *18*, 2143

**DOI:** 10.3390/ijms26104717

**Published:** 2025-05-15

**Authors:** Elisa Borsani, Barbara Buffoli, Veronica Bonazza, Russel J. Reiter, Rita Rezzani, Luigi F. Rodella

**Affiliations:** 1 Department of Clinical and Experimental Sciences, Division of Anatomy and Physiopathology, University of Brescia, Viale Europa 11, 25123 Brescia, Italy; elisa.borsani@unibs.it (E.B.); barbara.buffoli@unibs.it (B.B.); veronica.bonazza@unibs.it (V.B.); rita.rezzani@unibs.it (R.R.); 2 Interdipartimental University Center of Research “Adaption and Regeneration of Tissues and Organs—(ARTO)”, University of Brescia, Viale Europa 11, 25123 Brescia, Italy; 3 Department of Cell Systems and Anatomy, The University of Texas Health Science Center, San Antonio, TX 78229, USA; reiter@uthscsa.edu

In the original publication [1], there was an error in Figure 4. The same images appeared in two different immunohistochemical panels (Figures 3 and 4) of the article. This may have occurred due to an error during the file upload process. The corrected Figure 4 is shown below. The authors state that the scientific conclusions are unaffected. This correction was approved by the Academic Editor. The original publication has also been updated. The authors are grateful to the journal for its assistance and cooperation in resolving this issue.

## Figures and Tables

**Figure 4:**
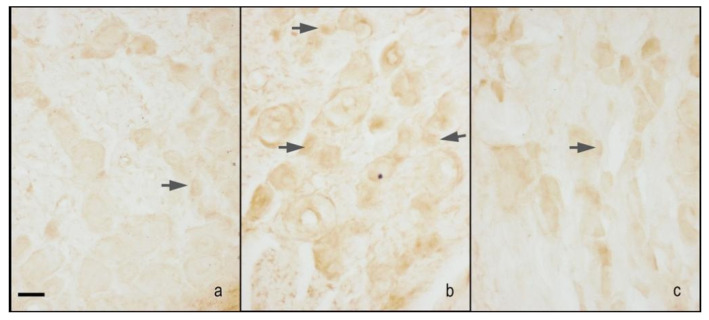
Microphotographs of iNOS immunostaining of the DRG. Microphotographs of iNOS immunostaining of the right DRGs of sham-operated rats treated with 1% ethanol in saline (vehicle for melatonin) (**a**), CCI animals treated with 1% ethanol in saline (vehicle for melatonin) (**b**), and CCI animals treated with melatonin 10 mg/kg (**c**). Arrows indicate small neurons. Bar = 40 μm.
